# Glycogen synthase kinase-3β: a promising candidate in the fight against fibrosis

**DOI:** 10.7150/thno.47717

**Published:** 2020-09-23

**Authors:** Hanxue Zheng, Zhi Yang, Zhenlong Xin, Yang Yang, Yuan Yu, Jihong Cui, Hongbo Liu, Fulin Chen

**Affiliations:** 1 Lab of Tissue Engineering, Faculty of Life Sciences, Northwest University, 229 TaiBai North Road, Xi'an 710069, China; 2 Provincial Key Laboratory of Biotechnology of Shaanxi, Northwest University, 229 TaiBai North Road, Xi'an 710069, China; 3 Key Laboratory of Resource Biology and Biotechnology in Western China, Ministry of Education. Faculty of Life Sciences, Northwest University, 229 Taibai North Road, Xi'an 710069, China; 4 Key Laboratory of Resource Biology and Biotechnology in Western China, Ministry of Education. School of Medicine, Northwest University, 229 Taibai North Road, Xi'an 710069, China

**Keywords:** Glycogen synthase kinase-3β, Fibrosis, Signaling pathway, Epithelial-mesenchymal transition, Aging

## Abstract

Fibrosis exists in almost all organs/tissues of the human body, plays an important role in the occurrence and development of diseases and is also a hallmark of the aging process. However, there is no effective prevention or therapeutic method for fibrogenesis. As a serine/threonine (Ser/Thr)-protein kinase, glycogen synthase kinase-3β (GSK-3β) is a vital signaling mediator that participates in a variety of biological events and can inhibit extracellular matrix (ECM) accumulation and the epithelial-mesenchymal transition (EMT) process, thereby exerting its protective role against the fibrosis of various organs/tissues, including the heart, lung, liver, and kidney. Moreover, we further present the upstream regulators and downstream effectors of the GSK-3β pathway during fibrosis and comprehensively summarize the roles of GSK-3β in the regulation of fibrosis and provide several potential targets for research. Collectively, the information reviewed here highlights recent advances vital for experimental research and clinical development, illuminating the possibility of GSK-3β as a novel therapeutic target for the management of tissue fibrosis in the future.

## Introduction

Fibrosis refers to the excessive formation of fibrous tissue associated with chronic inflammatory injury and is also a hallmark of the aging process. Tissue fibrosis occurs in almost all organs, mainly caused by ECM accumulation and parenchymal cell reduction, with increased expression of α-smooth muscle actin (α-SMA) and numerous ECM markers. Organ fibrogenesis has been acknowledged as the repair response of the body after an injury, which maintains the structural integrity of tissues and organs. However, excessive or uncontrolled repair processes can lead to organ fibrosis. In severe cases, the development of fibrosis disrupts normal structure and function and poses a serious threat to human health and life. In developed countries, fibrotic diseases account for nearly 45% of all deaths, but there is currently no effective method for tissue fibrosis prevention and treatment 1. Recent evidence suggests that many molecules play important roles in the development and progression of fibrosis. Among them, GSK-3β could be used as an important therapeutic target that cannot be ignored.

GSK-3 is a Ser/Thr-protein kinase with two isoforms, α and β, which are highly homologous in the kinase domain and are significantly different in their N- and C-terminal sequences 2, 3. In 1980, Woodgett first determined that GSK-3 acted as a rate-limiting enzyme in glycogen synthesis 4. Although the GSK-3 isoforms have some similarities in structures and overlapping functions, they still have many different properties 5. GSK-3α plays an essential and specific role in cardiomyopathy, fatty acid accumulation, insulin resistance, aging, and various cancers 6, 7, but it is rarely reported in fibrotic diseases. In this review, we mainly focus on GSK-3β. GSK-3β is a constitutively active kinase contributing to a variety of biological events, including embryonic development, cell differentiation, apoptosis, transcription, and translation 8-10. Studies have shown that GSK-3β plays an important role in the protection of initial inflammatory injury, activation of related effector cells, EMT process, and ECM accumulation by regulating many critical signaling pathways during the fibrotic process. Moreover, GSK-3β has received extensive attention as an essential regulator in aging and longevity. Thus, an in-depth understanding regarding the roles of GSK-3β in the fibrotic response may help find relevant targets for anti-fibrotic clinical therapeutics.

This review emphasizes the latest research advances regarding the roles of GSK-3β in fibrosis. First, we summarize the upstream regulatory factors of GSK-3β and its downstream targets in fibrogenesis. Next, we explain the role of GSK-3β in the fibrosis of various organs and tissues, such as the heart, lung, liver, and kidney. Moreover, we discuss the relationship between GSK-3β and ROS in fibrosis and the relationship between GSK-3β and aging-related fibrosis. We also focus on the clinical application of GSK-3β in fibrosis. Finally, we provide perspectives on the latest research and particularly discuss the differences in the effects of GSK-3β. Taken together, the information here should serve as a comprehensive reference for the identified roles of GSK-3β in fibrotic diseases, and it will helpfully become a novel therapeutic target for the management of tissue fibrosis.

## Upstream regulators of GSK-3β

**Figure 1:**
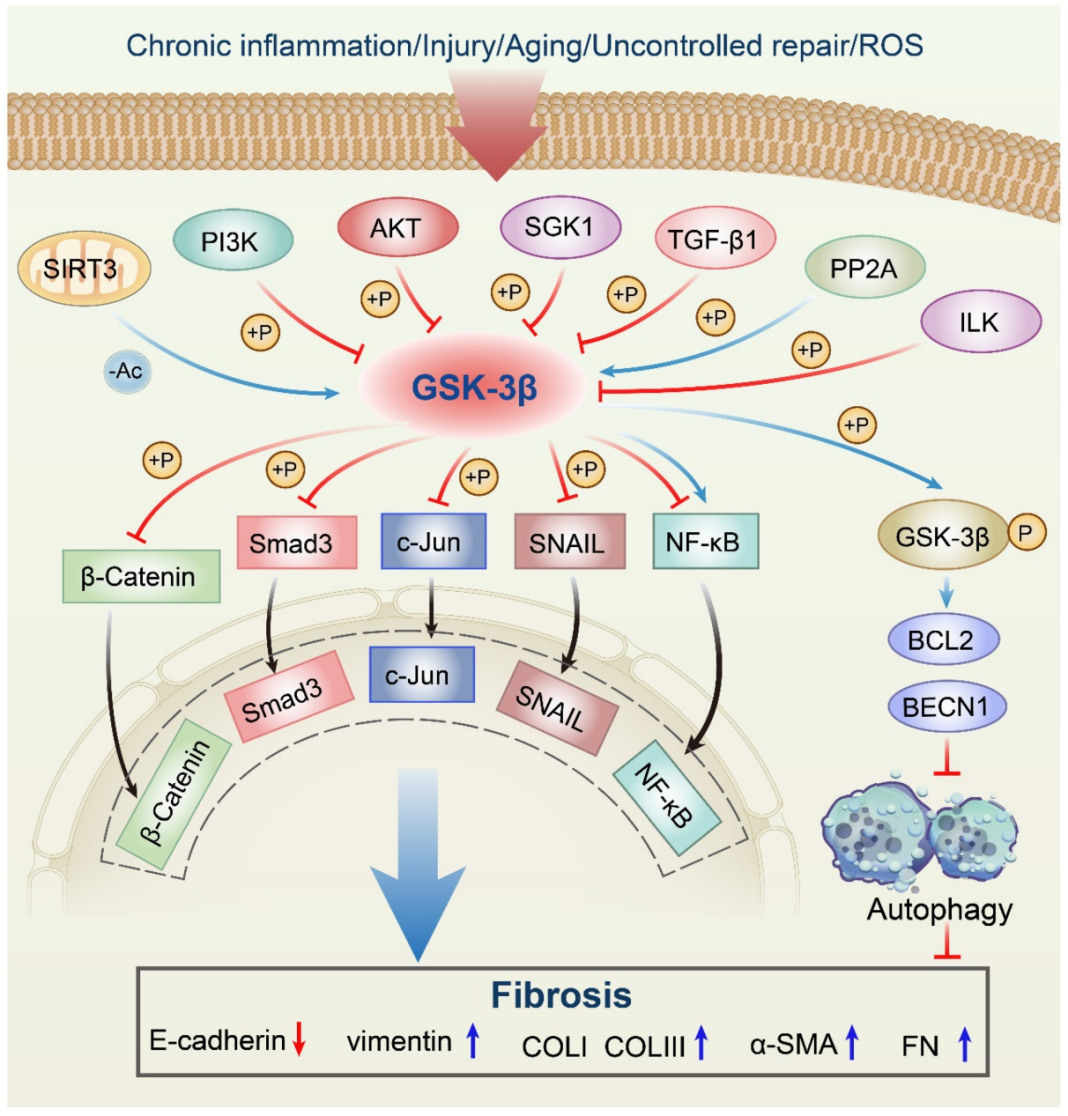
** Anti-fibrotic roles of the GSK-3β signaling network in fibrosis.** SIRT3 deacetylates GSK-3β in mitochondria, which results in the instability of substrates, such as SMAD3 and c-Jun, and reduces their import into the nucleus. The phosphorylation of GSK-3β at Ser9 by PI3K/AKT signaling pathway leads to its inactivation. SGK1 can inactivate GSK-3β by phosphorylation, thus contributing to the development of fibrosis. TGF-β1 induces α-SMA expression and triggers collagen production through increasing the expression of the Ser-9-phosphorylated inactive form of GSK-3β. The GSK-3β expression can be induced by PP2A, thereby limiting the proliferation of collagen. Activated ILK subsequently increases Ser9 GSK-3β phosphorylation, which is associated with many forms of adult fibrosis. Moreover, GSK-3β can mediate phosphorylation of substrates, including SNAIL, BCL2, β-Catenin, SMAD3, c-Jun, and NF-κB, which almost leads to the inhibition of these downstream effectors. ROS, reactive oxygen species; SIRT3, sirtuin 3; PI3K, phosphatidylinositol 3-kinase; Akt, protein kinase B; SGK1, serum and glucocorticoid-induced protein kinase 1; TGF-β1, transforming growth factor-β1; PP2A, protein phosphatase 2A; ILK, integrin-linked kinase; NF-κB, nuclear factor-kappaB; GSK-3β, glycogen synthase kinase-3β; BCL2, B-cell lymphoma 2; BECN1, Beclin1; Ac, acetylation; P, phosphorylation; α-SMA, α-smooth muscle actin; COLI, collagen I; COLIII, collagen III; FN, fibronectin.

In response to multiple upstream molecules, GSK-3β participates in several central intracellular signaling pathways, including oxidative stress, metabolism, cell proliferation, inflammation, and apoptosis. For instance, Sirtuin 3 (SIRT3) can deacetylate GSK-3β in mitochondria, which results in the instability of substrates, such as SMAD3 and c-Jun, and reduces their import into the nucleus 11. The phosphorylation of GSK-3β at Ser9 site by phosphatidylinositol 3-kinase (PI3K)/Protein kinase B (AKT) signaling pathway leads to its inactivation 12, 13. Serum and glucocorticoid-induced protein kinase 1 (SGK1) can also inactivate GSK-3β by phosphorylation, thus contributing to the development of fibrosis 14. In addition, transforming growth factor-β1 (TGF-β1) induces α-SMA expression and triggers collagen production through increasing the expression of the Ser-9-phosphorylated inactive form of GSK-3β 15, 16. The GSK-3β expression can be induced by protein phosphatase 2A (PP2A), thereby limiting the proliferation of collagen 17, 18. Moreover, activated integrin-linked kinase (ILK) subsequently increases Ser9 GSK-3β phosphorylation, which is associated with many forms of adult fibrosis 19. Since a comprehensive discussion on all upstream regulators is too verbose, we will focus on a couple of molecules already extensively studied in fibrotic diseases related to GSK-3β **(Figure 1, Table 1)**.

### SIRT3

SIRT3, a member of the NAD^+^-dependent class III deacetylase sirtuin family 20, is mainly localized to mitochondria, and its expression levels are regulated by caloric restriction and endurance exercise 21. SIRT3 activation has been demonstrated to reduce fibrosis in the heart 22, lung 23, kidney 24, and liver 25 fibrosis models. Many studies have shown that SIRT3 plays a crucial regulatory role as an upstream gene of GSK-3β in various cellular processes, including cellular metabolism, senescence, differentiation, and apoptosis 26-29.

It has been reported that SIRT3 knockout mice develop tissue fibrosis of multiple organs with age. Knockdown of SIRT3 in the cell caused hyperacetylation of GSK-3β at residue K5, resulting in impaired GSK-3β activity, thereby inhibiting its ability to phosphorylate substrates. Decreasing GSK-3β-induced phosphorylation results in the stabilization of its substrates, such as SMAD3 30 and c-Jun 31, increasing their import into the nucleus, where they regulate the expression of profibrotic genes and promotes the transformation of fibroblasts into myofibroblasts. Conversely, mitochondrial SIRT3 transgenic mice can further activate GSK-3β by reducing GSK-3β acetylation, resulting in decreased levels of TGF-β1 and SMAD3, ultimately blocking fibrosis 11.

Overall, GSK-3β acts as the downstream target of SIRT3, allowing SIRT3 to exert functions of controlling tissue aging and aging-related fibrotic remodeling.

### AKT

AKT is a Ser/Thr kinase involved in crucial cellular signaling pathways. The PI3K/AKT signaling pathway is widely present in cells and is involved in cell growth, proliferation, differentiation, and apoptosis. Moreover, the PI3K/AKT signaling pathway has been implicated in regulating GSK-3β 32, 33. AKT regulates the development of the fibrotic process by phosphorylating the Ser9 site of GSK-3β, which leads to its inactivation.

As an angiogenesis inhibitor, Mindin overexpression significantly attenuates cardiac hypertrophy, left ventricular dysfunction, and myocardial fibrosis, which is partly attributed to the further activation of GSK-3β by blocking AKT in a neonatal rat model of cardiac hypertrophy 34. In mouse lung fibroblasts, overexpression of phosphatase and tensin homolog deleted on chromosome 10 (PTEN) can significantly inhibit LPS-induced fibroblast proliferation, differentiation, and collagen secretion by inhibiting AKT phosphorylation and further activating GSK-3β 35. Similarly, inhibiting AKT activation and GSK-3β phosphorylation may attenuate EMT-related E-cadherin inhibition, α-SMA elevation, and morphological transformation into a myofibroblast-like phenotype, thereby ameliorating proteinuria-induced tubular EMT and ultimately reducing renal fibrosis 12.

### SGK1

SGK1, a member of the Ser/Thr-protein kinase family, was initially identified as an immediate-early gene whose mRNA levels increase sharply within 30 minutes when cells are exposed to serum or glucocorticoid (GC) 36. SGK1 has been demonstrated to play a critical role in regulating cell survival, proliferation, and differentiation 37. In the human body, overexpression of SGK1 is involved in the pathophysiology of various diseases, including hypertension, obesity, diabetes, stroke, fibrotic diseases, autoimmune diseases, and tumor growth 38. Recent studies have identified that SGK1 can inactivate GSK-3 by phosphorylation, thus contributing to the development of fibrosis and EMT.

A significant finding indicated that SGK1 is upregulated after deoxycorticosterone acetate (DOCA)/high salt treatment, further promoting the phosphorylation of GSK-3β and inhibiting GSK-3β activity, leading to severe fibrosis. Conversely, in SGK1-deficient mice, fibrosis is significantly attenuated after DOCA/high salt treatment 39. Voelkl *et al.* 40 constructed gene-targeted mice carrying the mutant GSK-3β in which the codon encoding Ser9 of the GSK-3β gene is altered to encode non-phosphorylatable alanine. The results confirmed that after unilateral ureteral obstruction (UUO), SGK1 is downregulated in the constructed mouse model and cannot phosphorylate GSK-3β-Ser9. At the same time, the expression of α-SMA, collagen I (COLI) and collagen III (COLIII) are significantly decreased, thereby suppressing obstruction-induced EMT and renal fibrosis 41.

In summary, these results strongly suggest that GSK-3β can significantly inhibit organ hypertrophy and fibrosis as a target of SGK1 **(Figure 1, Table 1).**

## Downstream Effectors of GSK-3β

As an irreplaceable central molecule in the fibrotic network, GSK-3β can mediate phosphorylation of substrates, including SNAIL 42, B-cell lymphoma 2 (BCL2) 13, β-Catenin 43, SMAD3 30, c-Jun 31, Nuclear factor erythroid 2-related factor 2 (Nrf2) 44, and Nuclear factor-kappaB (NF-κB) 45, which almost leads to the inhibition of these downstream effectors. For instance, GSK-3β exerts its anti-fibrosis effect by negatively regulating the stability and enzyme activity of SMAD3, and then inhibits the occurrence of fibrosis 46. Similarly, we will focus on the molecules of particular significance that may become promising new targets for the management and treatment of tissue fibrosis **(Figure 1, Table 2)**.

### SNAIL

SNAIL is a zinc finger transcription factor that was first discovered in Drosophila 14. GSK-3β binds to and phosphorylates SNAIL at two consensus motifs to dually regulate the function of this protein. Phosphorylation by GSK-3β at motif 1 results in the association of SNAIL with β-transducin repeat-containing protein (β-TrCP), leading to the degradation of SNAIL, while GSK-3β binds and phosphorylates SNAIL at motif 2 to induce the nuclear export of SNAIL 42, 47, 48. The GSK-3β/SNAIL pathway is known to regulate EMT 49, 50. The initially identified mechanism underlying EMT regulation revealed that GSK-3β suppression leads to the upregulation of SNAIL and the downregulation of E-cadherin 47.

A significant finding indicates that TGF-β1 signaling activates AKT and further inhibits GSK-3β, thereby inducing nuclear accumulation and transcriptional activation of SNAIL, leading to EMT and promoting fibrotic progression 51. Similarly, treatment with the GSK-3β-specific inhibitor SB216763 or siRNA gene knockout GSK-3β completely inhibits the nuclear accumulation of SNAIL and partially restores E-cadherin expression levels. Remarkably, GSK-3β is an endogenous inhibitor of SNAIL. In irradiated cells, phosphorylation of GSK-3β-Ser9 increased in a time-dependent manner, causing its dissociation from SNAIL and a significant elevation in protein levels and nuclear translocation of SNAIL, accompanied by an increase in the levels of α-SMA and vimentin, which suggested that the epithelial cells acquire a mesenchymal-like morphology 52. Additionally, Yoshino *et al.* 53 found that in a rat model of UUO, increased levels of inactive GSK-3β result in the upregulation of SNAIL mRNA and protein levels in renal tubular epithelial cells of the obstructed kidney, leading to severe renal fibrosis. Treatment with the GSK-3β-specific inhibitors lithium and TDZD-8 could result in the accumulation of SNAIL protein in the nucleus.

Taken together, these results strongly suggest that the accumulation of SNAIL by elevated levels of GSK-3β phosphorylation may be a potential new target for clinical applications of fibrosis.

### BCL2

BCL2 is one of the most important oncogenes. As an inhibitor of apoptosis, it lays the foundation for the study of cell death mechanisms 54.

The IL17A/PI3K/GSK-3β/BCL2 signaling pathway is involved in the development and progression of acute and chronic pulmonary fibrosis by regulating inflammatory responses and autophagy. IL17A can activate PI3K, which further inhibits the activity of GSK-3β by phosphorylating it at Ser9, subsequently attenuating the binding of GSK-3β to BCL2 and preventing the phosphorylation of BCL2 from inhibiting the ubiquitin-mediated degradation of BCL2. Thus, enhanced expression of BCL2 promotes the binding of BCL2 to Beclin1 (BECN1) to inhibit the activation of autophagy. When exposed to various stimuli, BCL2 could be isolated from BECN1, thus enhancing autophagic activity in cells 13. Consequently, the GSK-3β inhibitor SB216763 can promote the interaction of BCL2 with BECN1 by inhibiting the binding of BCL2 to GSK-3β, resulting in the relative inhibition of autophagy and ultimately the excessive accumulation of collagen and an increase in acute pulmonary fibrosis 55.

### β-Catenin

As a multifunctional protein, β-Catenin plays an essential role in the process of cell proliferation, differentiation, and apoptosis 56, 57. The transfer of β-Catenin into the nucleus after accumulation in the cytoplasm is an important step in causing fibrosis.

When the Wnt/β-Catenin pathway is not activated, activated GSK-3β phosphorylates the Thr41, Ser37 and Ser33 residues of β-Catenin and forms a complex with it, which is then ubiquitinated and degraded by the proteasome. Conversely, when the Wnt/β-Catenin pathway is activated, it can cause decomposition of the complex, and then β-Catenin is released from the complex and accumulates in the cytoplasm, eventually translocating into the nucleus 43, 58. As an essential component of the complex, the inhibition of GSK-3β activity by LiCl_2_ treatment can promote the translocation of β-Catenin to the nucleus. Active β-Catenin can induce the synthesis of α-SMA, COL1, and fibronectin (FN) 59-61. Stimulation of TGF-β1 induces the phosphorylation of p38 mitogen-activated protein kinase (MAPK), which phosphorylates GSK-3β-Ser9, to prevent phosphorylation of β-Catenin. Peroxisome proliferator-activated receptor γ (PPARγ) ligands can inhibit TGF-β1-induced p38 MAPK activity, which prevents GSK-3β phosphorylation, reduces the levels of active β-Catenin, and thus prevents the increased synthesis of profibrotic molecules, such as α-SMA, COL1 and FN 62.

A better understanding of the GSK-3β and β-Catenin signaling networks may provide a new therapeutic target in the process of fibrosis.

### Nrf2

Nrf2 is a cap'n'collar (CNC) basic-region leucine zipper (bZIP) nuclear transcription factor. Current evidence has demonstrated that GSK-3β manipulates nuclear exclusion and degradation of Nrf2 via the regulation of β-Trcp, which acts as a scaffolding protein for the Skp1-Cull-Rbx1/Roc1 ubiquitin ligase complex 63, 64.

GSK-3β knockout reinforces Nrf2 activation and nuclear accumulation, contributing to the anti-oxidant response. Conversely, GSK-3β overexpression blunts Nrf2 response and exacerbates cell injury, which could be abolished by treatment with SB216763 44. Treatment of diabetic nephropathy mice with zinc upregulates GSK-3β phosphorylation, resulting in a reduction in the export of Nrf2 to the cytosol, thereby ameliorating renal fibrosis 65. Moreover, polysaccharide isolated from okra (OP) may enhance the Nrf2 level in the nucleus via the inhibition of GSK-3β signal pathway in the type 2 diabetes mellitus (T2DM) mice, and this effect correlates with decreased liver fibrosis 66.

Given the limited research on the roles of GSK-3β/Nrf2 axis in fibrotic diseases, more attention should be focused on the elucidation of GSK-3β mechanisms in the regulation of Nrf2 of various classes in the future.

### NF-кB

NF-κB is a redox-sensitive transcriptional factor that regulates the expression of many genes involved in immune and inflammatory responses 67. In unstimulated cells, NF-κB is found in the cytoplasm and is bound to IκB proteins, which prevent sequester NF-κB complexes from entering the nuclei. When these cells are stimulated by oxidative stress and pro-inflammatory cytokines, The degradation of IκB proteins is triggered by the phosphorylation of IκB kinase (IKK) complex, releasing bound NF-κB dimers to translocate to the nucleus 68.

However, GSK-3β has been reported to both promote 69 and repress 70, 71 NF-κB activity. Atorvastatin (ATOR)-induced GSK-3β inhibition markedly suppresses NF-кB nuclear translocation and apoptosis, which alleviates fibrosis and cardiac dysfunction in high glucose (HG)-cultured cardiomyocytes and diabetic mice 72, 73. Correspondingly, individuals with low immune function, especially patients with cystic fibrosis, are susceptible to pulmonary infection with *Burkholderia cenocepacia* (*B. cenocepacia*), leading to a rapid decline in lung function. The investigations found that PI3K/AKT signaling inactivates GSK-3β after *B. cenocepacia* infection, thereby enhancing NF-κB activity but not NF-κB phosphorylation and proinflammatory cytokine production. Under *B. cenocepacia* infection, it appears that the inactivation of GSK-3β is the primary mechanism by which PI3K/AKT regulates NF-κB activity, which may be an essential factor leading to cystic fibrosis 45. This discrepancy might be attributed to the differences in the organ types and protocols.

The GSK-3β/NF-κB signaling pathway is a representative downstream pathway in GSK-3β mediated anti-fibrosis. However, further clarification of the specific mechanisms of this pathway may contribute to the therapeutic effect of fibrotic diseases **(Figure 1, Table 2).**

## Relationship between GSK-3β and ROS in fibrosis

Oxidative stress plays a crucial role in the pathogenesis of fibrosis. When multiple harmful stimuli trigger oxidative stress, excessive accumulation of reactive oxygen species (ROS) results in structural and functional damage to cells 74. Correspondingly, suppression of oxidative damage effectively inhibits or even reverses the fibrotic process in various animal models 75, 76. Acetaldehyde stimulates GSK-3β phosphorylation at Ser9 and promotes ROS accumulation, which exacerbates the fibrogenic pathway in human HSC 77. Glutathione S-transferase A3 (GSTA3) is regarded as an anti-oxidative protease, Chen *et al.* 78 discovered that GSTA3 suppresses intracellular ROS accumulation and further mitigates CCl_4_-induced fibrotic livers in rat models by activating GSK-3β, which results in potently blocked the expression of the α-SMA and FN. Besides, HG stimulates ROS production, leading to the inhibition of GSK-3β and the promotion of EMT process. Meanwhile, the expression level of epithelial cell marker E-cadherin is decreased, the mesenchymal markers vimentin and α-SMA are both promoted, as evidenced by induced renal fibrosis generation 42, 79. Moreover, knockdown of endogenous Nox2 significantly activates GSK-3β by inhibiting its phosphorylation in a rabbit CAVD model, along with significant attenuation of aortic valve ROS production and fibrosis 80. These findings reveal that ROS trigger GSK-3β inactivation in a manner dependent on Ser9 phosphorylation and subsequently activate the downstream signaling cascade to promote the process of fibrogenesis. Thus, GSK-3β signaling activation may represent a novel therapeutic target for fibrotic diseases caused by ROS.

## Roles GSK-3β plays in the fibrosis of multiple organs

### The anti-fibrotic roles of GSK-3β in the fibrosis

#### GSK-3β in myocardial fibrosis

Myocardial fibrosis is the common pathological manifestation of alterations in cardiac structure and function due to aging, mainly manifested by the proliferation of cardiac fibroblasts and excessive deposition of collagen in myocardial tissue 81. Myocardial fibrosis is closely related to cardiovascular diseases, such as cardiac hypertrophy, myocardial infarction (MI), dilated cardiomyopathy, and heart failure. The role of the GSK-3β in cardiomyocyte biology has been well established. However, *in vivo* studies supporting the role of GSK-3β family of kinases in myocardial fibrosis are still at the very early stages. Cardiac hypertrophy is characterized by a structural rearrangement of the cardiac chamber wall that is involved in cardiomyocyte hypertrophy and ultimately fibrosis. *In vivo* and *in vitro* experiments confirmed that piperine activates GSK-3β by blocking AKT activation, which consequently inhibits the conversion of neonatal rat cardiac fibroblasts to myofibroblasts, reduces α-SMA and collagen accumulation, and eventually alleviates cardiac hypertrophy and fibrosis. However, overexpression of AKT or knockdown of GSK-3β reverses the piperine-mediated protection of cardiac fibroblasts 82. Cathepsin L (CTSL) blocks cardiac hypertrophy and improves cardiac function by activating GSK-3β in rat neonatal cardiac myocytes. This effect correlates with reduced inflammation, increased collagen degradation and fibrosis levels, suggesting that GSK-3β exerts an anti-fibrosis effect in the process of fibrogenesis 83. It has been reported that 2,5-dimethylcelecoxib (DM-celecoxib) can inhibit myocardial fibrosis in mice with dilated cardiomyopathy by activating GSK-3β, which contributes to prolonged lifespan. Ai *et al.* 84 found that hearts in GSK-3β heterologous defect (GSK-3β^+/-^) mice showed more severe interstitial fibrosis than those in wild-type mice after transverse aortic constriction (TAC), suggesting that the decrease in GSK-3β activity aggravated cardiac hypertrophy. DM-celecoxib can control cardiac hypertrophy and myocardial fibrosis caused by TAC by activating GSK-3β. These results suggest that GSK-3β activators may provide a novel therapeutic strategy for the treatment of myocardial fibrosis and heart failure. Additionally, celastrol, a natural compound and nicotinamide adenine dinucleotide phosphate oxidase 2 (Nox2) inhibitor, can mitigate aortic valve fibrosis by upregulating GSK-3β gene expression in cultured porcine aortic valvular interstitial cells (AVICs) and the rabbit calcific aortic valve disease (CAVD) models, as evidenced by attenuated fibrotic marker FN 80.

The persistent inflammatory response after MI leads to myocardial fibrosis, which is characterized by myofibroblast hyperplasia, transformation, and collagen protein deposition. Hind *et al.* 81 demonstrated that GSK-3β knockout mice markedly triggers the transformation of fibroblasts to myofibroblasts and the accumulation of ECM, leading to a significant increase in fibrosis and scar formation, suggesting that GSK-3β is a vital inhibitor of myocardial fibrosis, especially in the remodeling stage after MI. Maintaining GSK-3β in an active state can provide therapeutic options for inhibiting fibrosis and limiting maladaptation remodeling. In a rat model of chronic heart failure after isoproterenol-induced MI, GSK-3β appears to be phosphorylated and inhibited at Ser9. Treatment with poly (ADP-ribose) polymerase (PARP) inhibitor (L-2286) reduces this intense phosphorylation to activate GSK-3β, which significantly reduces post-infarction cardiac hypertrophy and interstitial fibrosis, thereby ameliorating the progression of heart failure 85. Constitutively active GSK-3β prevents pathological hypertrophy in transgenic mice, which seems to be beneficial for the protection of myocardial fibrosis.

#### GSK-3β in pulmonary fibrosis

Idiopathic pulmonary fibrosis (IPF) is a progressive and fatal fibrotic interstitial pneumonia. The incidence of IPF is positively correlated with age and is increasing yearly 86. The main pathological features of IPF are attributed to repeated alveolitis, causing tissue damage and abnormal repair, which leads to fibroblast proliferation and excessive ECM deposition, and ultimately results in abnormal remodeling of the lung parenchyma 87, 88. Recent studies have revealed that GSK-3β plays a vital role in the abovementioned IPF pathological response. Inhibition of GSK-3β by LiCl_2_ increases normal fibroblast proliferation by approximately three-fold. Normal fibroblasts activate PP2A by enhancing integrin α2β1 expression and subsequently promote dephosphorylation and activation of its target matrix GSK-3β, leading to the inactivation of β-Catenin, which inhibits the pathological proliferation of fibroblasts and further collagen polymerization. In IPF fibroblasts, the expression of integrin α2β1 is abnormally low and cannot properly activate PP2A, thereby accumulating inactive GSK-3β and promoting β-Catenin activation and pathological proliferation. Blockade of α2β1 integrin in normal fibroblasts reproduces the IPF phenotype and gives rise to the inability of these cells to activate PP2A, subsequently resulting in high levels of phosphorylated GSK-3β and enhanced collagen accumulation 17. Filippo *et al.* 16 indicated that the treatment of human lung fibroblasts with a GSK-3β inhibitor (lithium chloride) increases β-Catenin translocation into the nucleus, promotes α-SMA expression and collagen production, and induces the phenotypic transformation of human lung fibroblasts into myofibroblasts. Moreover, high levels of secreted protein acidic and rich in cysteine (SPARC) are expressed in IPF fibroblasts, which mediate the activation of β-Catenin by activating AKT and inhibiting GSK-3β, leading to increased α-SMA expression and collagen accumulation 89. Recently, growing evidence has revealed that EMT is an essential pathway for fibrosis. In pulmonary fibrosis, EMT can be a new source of fibroblasts and a crucial contributor 90. Some studies found that activated GSK-3β inhibits EMT, as indicated by the decreased levels of vimentin and α-SMA and increased levels of E-cadherin, thereby alleviating pulmonary fibrosis 51, 91. These abovementioned roles played by GSK-3β may be important processes in inhibiting the development of IPF fibrosis.

#### GSK-3β in hepatic fibrosis

In normal liver, hepatic stellate cells (HSCs) are in a resting state with low proliferative activity, and their primary function is to store vitamin A 92, 93. When the liver is damaged by inflammation or mechanical stimulation, HSCs are activated and then transdifferentiated from a quiescent state to a myofibroblast phenotype, resulting in cell proliferation, α-SMA expression, collagen accumulation, ECM synthesis, and eventually hepatic fibrosis 94, 95. Studies have shown that GSK-3β is involved in HSC activation, transdifferentiation, and subsequent proliferative processes during liver fibrosis. Acetaldehyde elicits alcohol-induced liver fibrosis by activating human HSCs. The profibrotic role of acetaldehyde is mainly mediated by GSK-3β inactivation, which leads to the blockade of β-Catenin phosphorylation and degradation, thereby promoting the depolymerization of GSK-3β and β-Catenin and the transformation of β-Catenin into the nucleus and upregulation of fibrogenic pathway genes 96. Similarly, TGF-β1 induces ERK activation, leading to the inactivation of GSK-3β, followed by increased stability of β-Catenin, which contributes to HSC activation and liver fibrosis 15. Besides, IGF-1 and platelet-derived growth factor (PDGF) stimulate the proliferation of activated HSCs by inhibiting the pro-apoptotic effect of GSK-3β 97. Together, these findings demonstrate that inhibition of GSK-3β activity is involved in the activation of HSCs in the fibrosis process. Furthermore, thymosin beta-4 (Tβ4) acts as a regulator of HSC activation and is involved in a variety of biological processes 98. Treatment with carbon tetrachloride (CCl_4_) in Tβ4-overexpressing transgenic mice significantly upregulates the expression of linked protein kinase (ILK) and increases the phosphorylation of GSK-3β, which produced inactive GSK-3β, thereby promoting HSC activation and transdifferentiation and excessive collagen accumulation, ultimately resulting in liver fibrosis. The knockdown of Tβ4 in human HSCs downregulates the levels of ILK and pGSK-3β-Ser9, leading to the inactivation of HSCs. The above results indicated that Tβ4 inactivation reduces HSC activation and transdifferentiation at least partially via activating the GSK-3β pathway that inhibits liver fibrosis 99.

Christiane *et al.* 100 demonstrated that L-NAME, a nitric oxide (NO) biosynthesis inhibitor, increases collagen deposition by inhibiting GSK-3β activity, resulting in significant interstitial fibrosis. Another study revealed that excessive intake of fat is likely to induce a high risk of nonalcoholic fatty liver disease (NAFLD) development. Excessive lipid accumulation exacerbates liver inflammation and fibrosis. Through activating GSK-3β, actein treatment significantly reduces the levels of fibrosis markers, including α-SMA, COLI, and COLIII, thereby preventing the development of liver fibrosis in high-fat diet-induced mice 101.

#### GSK-3β in renal fibrosis

Renal fibrosis is the ultimate common manifestation of almost all types of kidney disease 102. Under continuous and severe injury, the transformation of fibroblasts and epithelial cells to myofibroblasts causes accumulation of ECM in the kidney, leading to renal fibrosis and eventually to end-stage renal failure 103. In the UUO mouse model, fingolimod (FTY720) downregulates the levels of α-SMA and collagen, prevents the formation of myofibroblasts, and reduces the synthesis of ECM protein by activating GSK-3β to repress the progression of renal fibrosis 104. LiCl_2_ has been shown to inhibit GSK-3β activity and increase the expression and activation of SNAIL and β-Catenin in cultured kidney epithelial cells. Knockout of AKT prevents UUO-induced EMT and renal fibrosis by increasing GSK-3β activity and decreasing SNAIL and β-Catenin expression 60. These results demonstrate that activation of GSK-3β can effectively alleviate renal fibrosis in UUO mouse models. Long-term exposure to lithium is associated with chronic interstitial fibrosis and may lead to its progression to end-stage renal disease. Lithium-induced GSK-3β inhibition is important in the profibrotic process. In addition, β-Catenin is a critical transcription factor involved in EMT, as a downstream marker of GSK-3β is increased with lithium treatment and partially reduced with coadministration of amiloride 59. Also, angiotensin II (ANG II)-dependent inhibition of GSK-3β activity and upregulation of β-Catenin expression induce the expression of fibrotic factors to augment renal tubulointerstitial fibrosis 105. These results confirmed that the activation of GSK-3β reduces the expression of β-Catenin to inhibit or slow the occurrence of renal fibrosis. In urinary protein (UP)-incubated rat renal tubulointerstitial cells, upregulation of soluble epoxide hydrolase (sEH) and inhibition of GSK-3β significantly stimulate EMT, decrease E-cadherin expression, and increase α-SMA expression and morphological conversion to a myofibroblast-like phenotype. However, pretreatment with the sEH inhibitor AUDA partially inhibits UP-induced phosphorylation of GSK-3β and increases GSK-3β activity. Similarly, pharmacological inhibition of PI3K with LY294002 reduces the phosphorylation of GSK-3β, thereby attenuating the levels of EMT markers 12. Moreover, Cheng *et al.* 37 verified that low expression of SGK1 leads to phosphorylated GSK-3β in normal kidneys, and the increase in GSK-3β activity reduces the accumulation of SNAIL, thereby significantly inhibiting obstruction-induced EMT and renal fibrosis. Meanwhile, α-SMA expression and collagen accumulation are also reduced. However, in UUO mice, stimulation of SGK1 expression and activation results in a decrease in GSK-3β activity, further inhibiting phosphorylation and degradation of SNAIL, thereby inhibiting E-cadherin transcription and ultimately promoting EMT. Additionally, high circulating follicle‐stimulating hormone (FSH) levels inactivate GSK‐3β and exacerbate tubulointerstitial fibrosis, correlated with increased the transcriptional and protein expression of profibrotic mediators (Collagen IV, FN, and PAI‐1) 106. Taken together, GSK-3β is closely related to the occurrence and development of renal fibrosis by affecting EMT and metabolic function.

#### GSK-3β in the fibrosis of other organs

**Figure 2:**
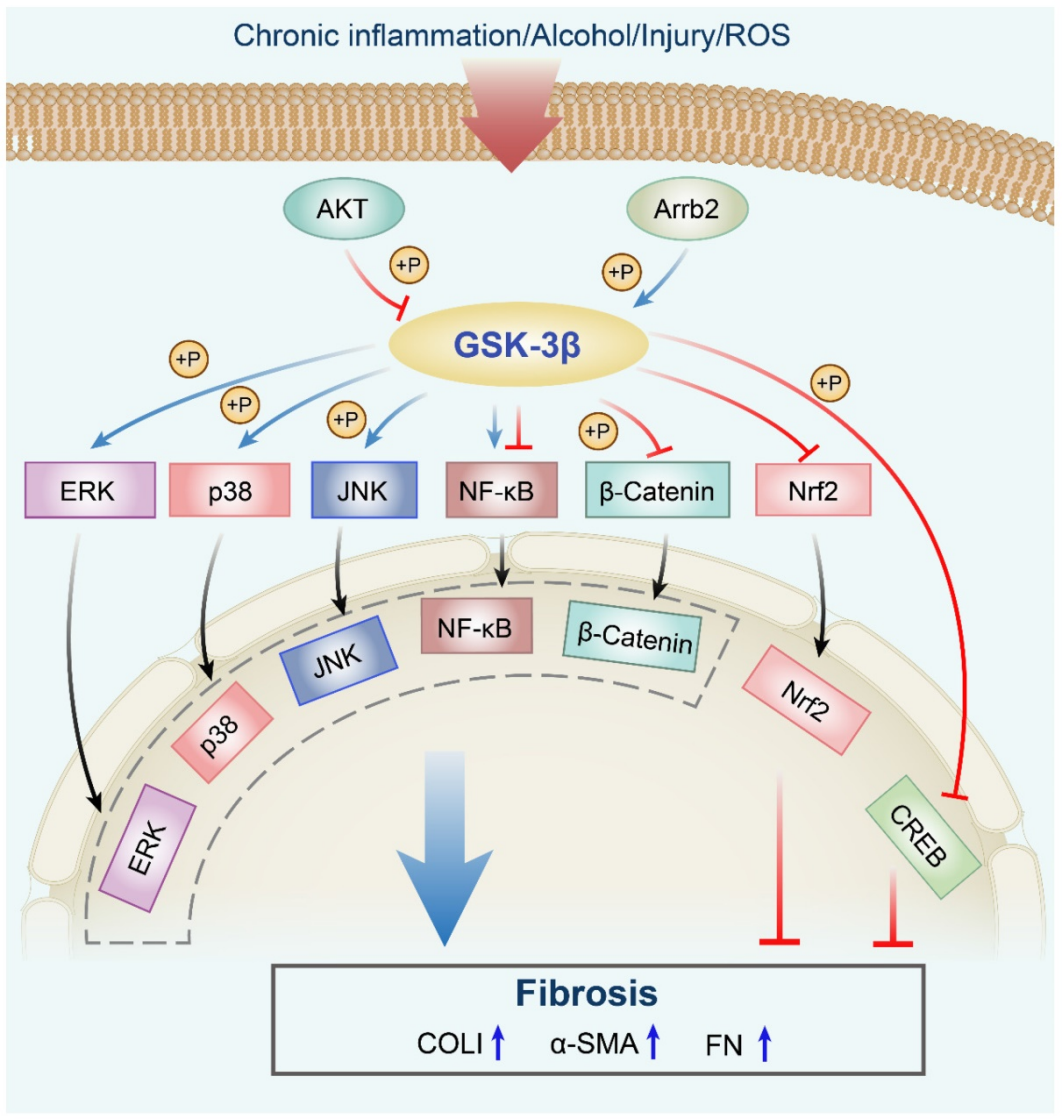
** Pro-fibrotic roles of the GSK-3β signaling network in fibrosis.** The AKT signaling phosphorylates GSK-3β at Ser9, which leads to its inactivation. Arrb2 knockout attenuates fibrosis through decreasing (by increased phosphorylation) GSK-3β activity. GSK-3β phosphorylates MAPK kinases, including p38, ERK, and JNK, which are implicated in fibrogenesis. Inhibition of the GSK-3β signaling pathway enhances the Nrf2 level in the nucleus. GSK-3β regulates the nuclear translocation of NF-κB to promote or inhibit its activity. Moreover, GSK-3β inhibition increases the phosphorylation of CREB at Ser133. ROS, reactive oxygen species; Akt, protein kinase B; Arrb2, β-arrestin 2; GSK-3β, glycogen synthase kinase-3β; ERK, extracellular regulated protein kinase; JNK, c-Jun N-terminal kinase; NF-κB, nuclear factor-kappaB; Nrf2, nuclear factor erythroid 2-related factor 2; GSK-3β, glycogen synthase kinase-3β; CREB, cAMP response element-binding protein; P, phosphorylation; α-SMA, α-smooth muscle actin; COLI, collagen I; FN, fibronectin.

Keloid is a fibroproliferative disorder characterized by fibroblast proliferation and excessive collagen deposition 107. Cai *et al.* 108 proved that activation of GSK-3β leads to decrease proliferation and increase apoptosis of keloid fibroblasts, thereby inhibiting the keloid fibrosis phenotype. A prominent feature of peritoneal dysfunction is the EMT of human peritoneal mesothelial cells (HPMCs). GC is one of the most commonly used as an anti-inflammatory drug that inhibits the deposition of ECM. In TGF-β1-stimulated HPMCs, GC improves the effect on peritoneal EMT by activating GSK-3β and inhibiting SNAIL, further inducing the mesenchymal-to-epithelial transition (MET) of mesothelial cells during phenotypic transformation. Therefore, activation of GSK-3β may be an important way to treat EMT and peritoneal fibrosis in peritoneal mesothelial cells 109. Intestinal fibrosis is characterized by the overproduction of activated myofibroblasts and accumulation of ECM proteins. In a mouse model of dextran sulfate sodium (DSS)-induced intestinal fibrosis, GED-0507-34 Levo (GED) treatment improves chronic colitis and fibrosis. Phosphorylation of GSK-3β-Tyr216 is significantly increased in DSS mice treated with GED compared to control and untreated mice, accompanied by decreased expression of α-SMA, COLI/III and FN, which indicated that GSK-3β acts as an EMT inhibitor in the intestine. Therefore, it can be inferred that GSK-3β activation may be necessary for GED treatment to exert a therapeutic effect in intestinal fibrosis by regulating the EMT process 110. Radiation-induced injury is common in patients with advanced cancer. Radiation causes injury to epithelial cells, which transforms into a mesenchymal phenotype, resulting in the accumulation of collagen and ECM, ultimately resulting in radiation fibrosis. Irradiated rat epithelial cells have a mesenchymal phenotype, in which GSK-3β is significantly inactivated, and the expression levels and nuclear translocation of SNAIL are elevated 52. Another study has shown that baicalin treatment reduces the induction of EMT induced by irradiation by activating GSK-3β 111. These above results suggested that inhibition of GSK-3β leads to mesenchymal morphology acquisition of the epithelial structure, and thus, GSK-3β is essential for maintaining the epithelial structure of the cells to inhibit fibrotic progress **(Figure 2, Table 3)**.

### The pro-fibrotic roles of GSK-3β in the fibrosis

Diabetic cardiomyopathy (DCM) is the leading cause of death in patients with diabetes, resulting in 50-80% of diabetes-related deaths. Among them, myocardial fibrosis plays a vital role in the development of DCM. Activated GSK-3β in diabetic rats reduces diastolic and systolic myocardial function and aggravates cardiac hypertrophy, correlated with excessive collagen accumulation. Berberine treatment ameliorates fibrosis and improves myocardial function by reducing GSK-3β activation in type 2 diabetic rats and palmitate-induced hypertrophic H9c2 cardiomyocytes, and this effect correlates with reduced collagen deposition to the same levels as healthy animals 112. Further studies verified that transgenic mice overexpression cardiac-specific metallothionein (MT-TG) are highly resistant to diabetes-induced cardiomyopathy. MT-TG significantly inhibits GSK-3β activity and ameliorates fibrosis in diabetic mice, followed by attenuated lipid accumulation, inflammation, oxidative damage, and remodeling. Additionally, treatment with the GSK-3β-specific inhibitor SB216763 also significantly represses diabetes-induced cardiac fibrosis by reducing cardiac lipid accumulation, cardiac inflammation, and nitrosation damage 113. These results indicated that GSK-3β inactivation could attenuate diabetes-induced cardiac glucose and lipid metabolism changes, inflammation, and fibrosis. Among the complications of diabetes, fibrosis is primarily ameliorated by inhibition of GSK-3β activity, which may be related to its involvement in glycogen anabolism.

In addition, Liu *et al.* 114 indicated that activation of the GSK-3β occurs during a chronic liver injury in mice. Morin (3, 5, 7, 2′, 4′-pentahydroxyflavone), a natural bioflavonoid, inhibits HSC activation by directly downregulating GSK-3β gene expression, resulting in attenuated liver fibrosis 115. In the bile duct ligation (BDL) mice, β-arrestin 2 (Arrb2) knockout enhances survival and attenuates hepatic injury and fibrosis through decreasing GSK-3β activity as well as reducing hepatocyte apoptosis 116. Additionally, the treatment of mice with chronic alcohol activates GSK-3β and aggravates renal fibrosis and function impairment. Meanwhile, α-SMA secretion, COLI, and FN deposition are also promoted. Importantly, inhibition of GSK-3β using SB216763 or TDZD-8 completely alleviates fibroblast activation and fibrosis development following renal ischemia-reperfusion injury (IRI) in mice 117, 118. Tanshinone IIA blunts GSK-3β overactivity and subsequent hyperactivation of MAPK pathways, which attenuates renal fibrogenesis and inflammation in murine models of AKI induced by IRI. However, these effects are reversed by sodium nitroprusside, a GSK-3β activator 119. Moreover, the administration of human lung fibroblasts with SB216763 inhibits GSK-3 and attenuates pulmonary fibrosis, correlated with prevented α-SMA and FN 120. GSK-3β inhibitor 9ING41 attenuates lung injury progression and pleural fibrosis in a mouse model of *Streptococcus pneumoniaee*-induced empyema by reducing Tyr-216 phosphorylation of GSK-3β, which results in reduced α-SMA and COLI, and blocked induction of mesothelial mesenchymal transition 121 (**Figure 2, Table 4**).

## Relationship between GSK-3β and fibrosis in aging

With the aging of the global population, the health problems caused by aging and related diseases have become increasingly prominent. One of the key factors reported to increase life expectancy is calorie restriction, which is primarily achieved by reducing dietary calorie intake 122, 123. High-fat diets (HFDs) can cause fibrosis in different organs and organ dysfunction during aging. Recent studies have found that HFD could induce cardiac hypertrophy and myocardial fibrosis in mice. The HFD group had significantly increased phosphorylation of GSK-3β and decreased expression of total GSK-3β, indicating that chronic HFD significantly inhibited GSK-3β activation and further increased β-Catenin expression and YAP nuclear translocation, thereby inducing cardiac hypertrophy and myocardial fibrosis. However, the intermittent fasting group did not show inhibited GSK-3β expression and thus did not induce myocardial fibrosis 124. The experimental results may suggest that GSK-3β has a protective effect on myocardial fibrosis in a HFD setting. Similarly, ELAINE *et al.* 125 demonstrated that a high-fat diet promoted cardiac remodeling and increased interstitial fibrosis. The level of GSK-3β protein was decreased in HFD mouse hearts, accompanied by increased expression of miRNA-21a, miRNA-29c, miRNA-144, and miRNA-195a. Bioinformatic prediction analysis indicated that GSK-3β is a potential target for miRNA-21a, miRNA-29c, miRNA-144, and miRNA-195a, which significantly attenuated transverse cardiomyocyte diameter and interstitial fibrosis. Moreover, calorie restriction can also protect cells and improve brain structure and memory 126. Nagalingam *et al.* 11 found that dietary restriction could delay the aging process and activate SIRT3, which further inhibited TGF-β1 signaling and controlled the conversion of fibroblasts to myofibroblasts by activating GSK-3β, thereby inhibiting fibrosis. In contrast, SIRT3 knockout mice develop tissue fibrosis in multiple organs with age. In SIRT3-deficient cells, GSK-3β was acetylated, thereby leading to the inhibition of its ability to phosphorylate substrates, ultimately increasing profibrotic gene expression and enhancing the transformation of fibroblasts into myofibroblasts. In summary, by activating GSK-3β, calorie restriction can control tissue aging and aging-related fibrosis remodeling. Therefore, GSK-3β can be used as a new target to delay aging and aging-related fibrosis.

## Clinical studies of GSK-3β in fibrosis

GSK-3β represents a signaling center at the intersection of multiple pathways implicated in fibrogenesis. Xiao *et al.* 127 demonstrated that enrolled 25 systemic sclerosis (SSc) patients (20 newly diagnosed and 5 who underwent pirfenidone treatment for 6 months) and 10 healthy controls. Lung tissues are obtained by open-chest surgery, the levels of GSK-3β phosphorylation are significantly lowered in the treatment of primary human lung fibroblasts from SSc patients with pirfenidone. However, treatment with the specific inhibitor AR-A14418 aggravates the fibrotic phenotype of SSc fibroblasts via inhibition of GSK-3β 31. Thus, the anti-fibrotic effects of pirfenidone in SSc patients are at least partly achieved by activation of GSK-3β. Furthermore, recent studies have revealed that osteopontin (OPN) can promote the fibrotic process in various organs, which is achieved through inhibition of GSK-3β signaling pathway and suppression of autophagic degradation of ECM in the circulation of atrial fibrillation (AF) patients, correlated with increased production of COLI and FN and the proliferation of fibroblasts 128. Moreover, it has been reported that miR-154-induced GSK-3β inhibition promotes myocardial fibrosis in human cardiac fibroblasts from 51 patients with cardiomyopathy. This action is reversed by the transfection of GSK-3β overexpression vector, suggesting that GSK-3β is a vital mediator for myocardial fibrosis 129. Indeed, we could not find any registered clinical trials of fibrotic diseases with GSK-3β inhibitors. However, we still believe that with the in-depth understanding of the GSK-3β signaling network, there will be more preclinical studies to explore many new drug combinations with GSK-3β inhibitors so as to take GSK-3β inhibitors into the clinic for the management of fibrotic diseases.

## Perspectives

Some of the latest research related to the GSK-3β signaling pathway provides potentially valuable strategies for the prevention and treatment of fibrotic diseases in the future. Circular RNA (circRNA) is a class of covalently closed endogenous RNA molecules formed by unique splicing mechanisms widely found in various life forms, such as plants and mammals. In recent years, it has been found that circRNAs function as miRNA sponges, which can competitively adsorb and bind miRNA and then participate in gene expression and regulation. Studies have shown that circRNA is closely related to various diseases, such as fibrotic diseases, tumors, and diabetes, and is expected to become a useful clinical diagnostic marker in the future. Notably, in osteosarcoma (OS), p-AKT expression decreased after knockout of hsa_circ_0007534, and the expression trend of p-GSK-3β was consistent with p-AKT. Therefore, the AKT/GSK-3β pathway could be activated by hsa_circ_0007534 to facilitate OS progression 130. However, the role of circRNA and GSK-3β has not been reported in fibrotic diseases, and it has also provided direction for the discovery of treatments for related diseases and new targeted drugs. Additionally, recent studies have shown that abnormal expression of long noncoding RNAs (lncRNAs) plays a crucial role in carcinogenesis and cancer metastasis. The knockout of H19 severely inhibits the activation of β-Catenin and GSK-3β, accompanied by the upregulation of epithelial markers (E-cadherin and ZO-1) and downregulation of mesenchymal markers (E-cadherin and vimentin), thereby regulating the EMT process. Overexpression of EZH2 and downregulation of H19 completely reversed H19 silencing-mediated inhibition of β-Catenin and GSK-3β activation in tongue cancer cells 131. It is worth noting that GSK-3β plays a vital role in the process of fibrosis by regulating EMT. Therefore, it is crucial to explore whether lncRNAs can regulate the GSK-3β mediated EMT process to play an anti-fibrotic role.

GSK-3β has been acknowledged as an essential regulator of fibrosis and maintaining GSK-3β in an active state can effectively inhibit fibrosis. However, GSK-3β still has unfavorable effects in the treatment of fibrosis due to the various pathological features produced by different organs. The reasons why GSK-3β exhibits different effects may be as follows: First, we have noticed that GSK-3β can produce disparate responses related to the different stress conditions, the severity of diseases, or the pre-environment of cells from diverse origins *in vivo*. Therefore, confirming other factors such as cell/tissue collection and maintenance will help to elucidate the profibrotic role of GSK-3β better. Second, only a small part of total cellular GSK-3 binds axin, casein kinase and APC in the cytosolic destruction complex to regulate the level of β-Catenin. In contrast, other GSK-3 cell pools have the opposite function by regulating Nrf2, NF-κB, and other transcription factors, as previously introduced 120. GSK-3 compartmentalization in the fibrotic response further explains that GSK-3β may play different functional roles in distinct intracellular signaling pathways or under different experimental conditions. However, the exact mechanism of the GSK-3β signaling network is unclear and needs further study. Third, *in vitro* and *in vivo* experiments have shown that GSK-3β inhibitors, such as TDZD-8 and 9ING41, can reduce the inflammatory response of organs and further delay the progression of these diseases 121. However, the inhibitor mechanisms may be significantly different (Table 1). For instance, pharmacological GSK-3β inhibitor SB216763 is a highly selective small-molecule inhibitor, which has been widely used to study the role of GSK-3β in related fibrotic diseases both *in vivo* and *in vitro* 116. It mainly controls the process of autophagy and apoptosis by regulating the downstream effectors of GSK-3β 116, 132. Additionally, 9ING41 and TDZD-8 both increase Ser-9 phosphorylation to inhibit GSK-3β activity. However, 9ING41 effectively reduced Tyr-216 phosphorylation. Further, 9ING41 is unlikely to off-target effects and is better tolerated 121. Further studies are needed to determine potential advantages and clinical applicability through toxicology analyses, dosing, and formulation optimization. Therefore, GSK-3β serves as a point of convergence for multiple fibrosis pathways downstream of diverse disease signals; animal models should be used to further study the effectiveness of GSK-3β inhibitors in fibrosis reactions. Moreover, research on the crosstalk will be more conducive to a deep understanding of the internal relationship between the GSK-3β pathway and fibrosis reactions in the future.

## Conclusion

The pathogenesis of fibrosis is complex, and there is still no effective treatment, which is a global problem that threatens human health. Current research on the involvement of GSK-3β in the pathogenesis of fibrosis has made some progress. When tissues are injured, GSK-3β can inhibit a series of downstream target genes, including SNAIL, BCL2, and β-Catenin. Thereafter, GSK-3β significantly attenuates the levels of α-SMA and vimentin, reduces collagen synthesis and accumulation, and thus inhibits fibrosis. Furthermore, GSK-3β can be modulated by a variety of proteins, such as SIRT3, AKT, and SGK1, to participate in various cellular activities.

However, GSK-3β plays different roles in various organs, diverse pathological stages, or experimental conditions. The abnormal expression of GSK-3β is not attributed to a single upstream pathway but to multichannel cross-regulation pathways that lead to fibrosis. In addition, the etiologies of fibrosis are diverse. It is impossible to understand its mechanism from unilateral studies systematically. Therefore, a single pathway of concern does not fully explain a certain pathogenic mechanism, and it requires an overall omics analysis and verification. As an irreplaceable regulator of fibrosis, a better understanding of the GSK-3β signaling network can provide promising new targets for the management and treatment of tissue fibrosis.

## Figures and Tables

**Figure 3:**
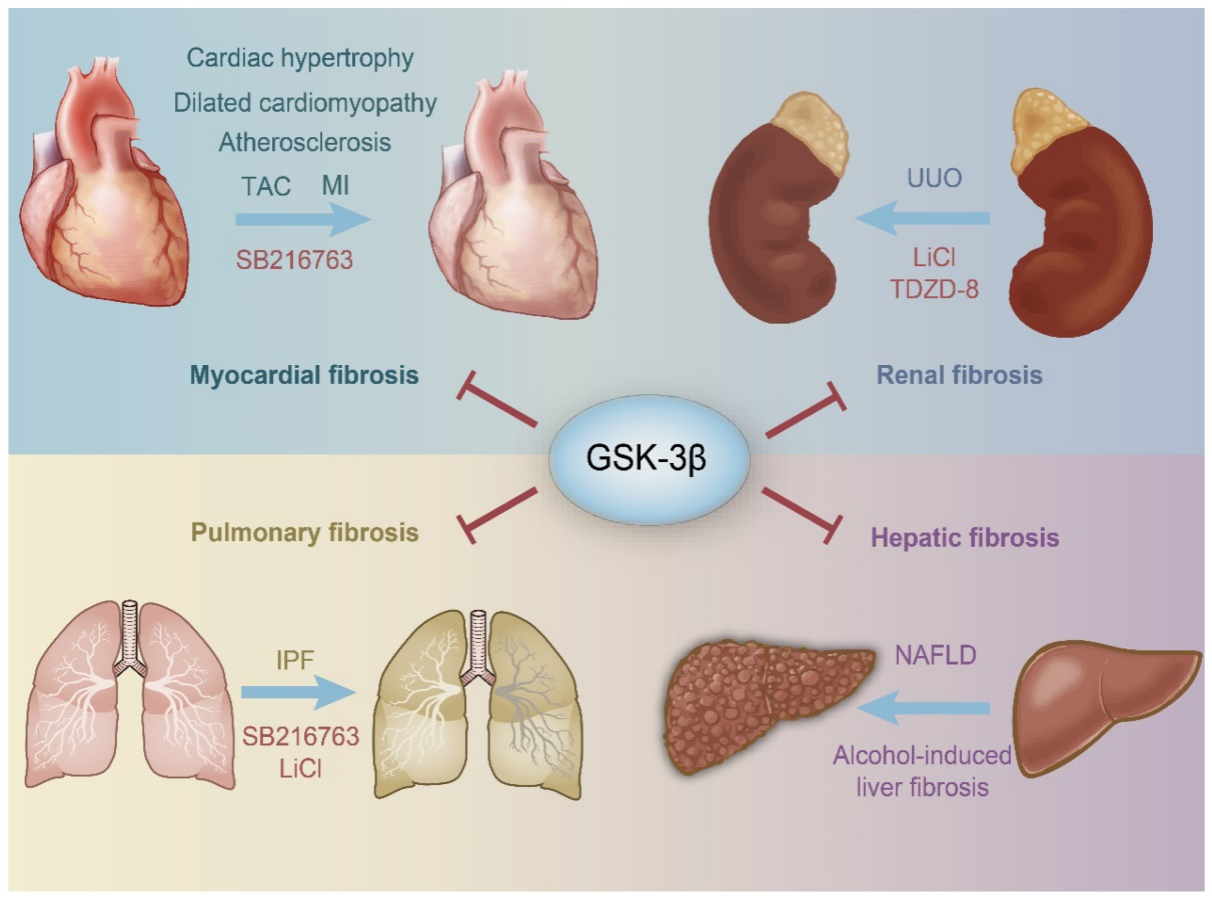
** Schematic representation of the protective role of GSK-3β in organ fibrosis.** GSK-3β inhibits the harmful stimuli to prevent the heart, kidney, lung, kidney, and liver from fibrosis. SB216763, LiCl and TDZD-8 are all GSK-3β inhibitors. TAC, transverse aortic constriction; MI, myocardial infarction; IPF, idiopathic pulmonary fibrosis; NAFLD, nonalcoholic fatty liver disease; UUO, unilateral ureteral obstruction; LiCl, Lithium Chloride.

**Table 1:** Effects of upstream modulators/molecules of GSK-3β in fibrosis.

Upstream modulators/molecules	Effects	Mechanisms	References
SIRT3	Activation	SIRT3 deacetylates GSK-3β at mitochondria.	11
AKT	Inhibition	AKT phosphorylates GSK-3β at Ser9.	12
SGK1	Inhibition	SGK1 phosphorylates GSK-3β at Ser9.	14
PI3K	Inhibition	PI3K phosphorylates GSK-3β at Ser9.	13
TGF-β1	Inhibition	TGF-β1 phosphorylates GSK-3β at Ser9.	133
PP2A	Activation	PP2A phosphorylates GSK-3β at Ser9.	17
ILK	Inhibition	ILK phosphorylates GSK-3β at Ser9.	19
Arrb2	Activation	Arrb2 phosphorylates GSK-3β at Ser9.	118
SB216763	Inhibition	SB216763 is a potent, selective and ATP-competitive GSK-3β inhibitor.	51, 134
Lithium Chloride	Inhibition	Lithium Chloride is a direct, non-competitive GSK-3β inhibitor.	16, 59
TDZD-8	Inhibition	TDZD-8 is a synthetic chemical, non-ATP competitive GSK-3β inhibitor.	53
9ING41	Inhibition	9ING41 inhibits GSK-3β activity through reduced Tyr216 phosphorylation.	121
CHIR99021	Inhibition	CHIR99021 is a potent and selective GSK-3β inhibitor.	135
AR-A14418	Inhibition	CHIR99021 is a selective and ATP-competitive GSK-3β inhibitor.	31

**Table 2:** Effects of downstream effectors of GSK-3β in fibrosis.

Downstream effectors	Mechanisms	References
SMAD3	GSK-3β phosphorylates SMAD3 at Thr66, inducing SMAD3 ubiquitination and degradation.	30
c-Jun	GSK3β phosphorylates c-Jun at the c-terminal Thr239, promoting c-Jun degradation.	31
SNAIL	Phosphorylation by GSK-3β at motif 1 results in the association of SNAIL with β-Trcp leading to the degradation of SNAIL, while GSK-3β binds and phosphorylates SNAIL at motif 2 to induce the nuclear export of SNAIL.	42
BCL2	Inhibition of GSK-3β activity attenuates the binding of GSK-3β to BCL2 and then prevents the phosphorylation of BCL2 from inhibiting the ubiquitination degradation of BCL2.	13
β-Catenin	The activated GSK-3β phosphorylates the Thr41, Ser37, and Ser33 residues of β-Catenin and forms a complex with it, which is then ubiquitinated and degraded by proteasomes.	43
Nrf2	The inhibition of GSK-3β signaling pathway enhances the Nrf2 level in the nucleus.	66
NF-κB	GSK-3β can regulate the nuclear translocation of NF-κB to promote or inhibit its activity.	72
MAPKs	GSK-3β can phosphorylate MAPK kinases, including p38, ERK, and JNK.	119
CREB	GSK-3β inhibition increases the phosphorylation of CREB at Ser133.	120

**Table 3:** Anti-fibrotic effects of GSK-3β in various organs/tissues.

Models	Organs/Tissues	Outcomes	References
C57BL/6 mice	Heart	Piperine treatment inhibits the conversion of cardiac fibroblasts to myofibroblasts, reduces α-SMA and collagen accumulation, and eventually alleviates cardiac hypertrophy and fibrosis.	82
Cardiac fibroblasts isolated from SD neonatal rats	Heart	SDT attenuates myocardial fibrosis by phosphorylating and activating GSK-3β at Tyr216. Meanwhile, α-SMA and COLI/COLIII are both decreased.	136
GSK-3β KO mice	Heart	Maintaining GSK-3β in an active state can inhibit fibrosis and limit maladaptation remodeling by transforming fibroblasts into myofibroblasts and accumulating ECM.	81
Human lung fibroblasts	Lung	In IPF fibroblasts, inactive GSK-3β promotes active β-catenin and pathological proliferation, and further enhances the pathological proliferation of fibroblasts and collagen polymerization.	17
Human adult lung fibroblasts	Lung	GSK-3β inhibitor promotes α-SMA expression and collagen production, and further induces the phenotypic transformation of human lung fibroblasts into myofibroblasts, leading to the pathophysiology of pulmonary fibrosis.	16
Male BALB/c mice	Lung	CIP4 silencing can effectively relieve diabetic pulmonary fibrosis through activation of GSK-3β, resulting in decreased levels of vimentin and α-SMA and increased levels of E-cadherin.	91
Human HSC line	Liver	Tβ4 inactivation inhibits liver fibrosis at least partially via activating the GSK-3β pathway, leading to the inhibition of HSC activation, transdifferentiation and collagen excessive accumulation.	99
C57BL/6 mice	Liver	GSK-3β activation reduces the levels of fibrosis markers, including α-SMA, COLI, and COLIII, thereby preventing the development of liver fibrosis in high fat diet-induced mice.	101
C57BL/6 mice	Kidney	Fingolimod downregulates the levels of α-SMA and collagen, prevents the formation of myofibroblasts, and reduces the synthesis of ECM protein by activating GSK-3β to repress the progression of renal fibrosis.	104
Rat renal proximal tubular epithelial cells	Kidney	UP incubation decreases E-cadherin expression, increases α-SMA expression, and the promotes morphological conversion to myofibroblast-like phenotype. However, sEH inhibitor AUDA treatment inhibits GSK-3β phosphorylation, thereby ameliorating EMT and renal fibrosis.	12
SGK1 KO mice	Kidney	Increased GSK-3β activity reduces α-SMA expression and collagen accumulation, thereby significantly inhibiting obstruction-induced EMT and renal fibrosis.	37
Human peritoneal mesothelial cells	Peritoneum	Activation of GSK-3β improves the effect on EMT and peritoneal fibrosis by inhibiting the α-SMA expression and restoring E-cadherin expression.	109
C57BL/6 mice	Intestine	GSK-3β activation significantly decreases the expression of α-SMA, COLI/III, and FN, further regulating the EMT process to exert a therapeutic effect in the intestine fibrosis.	110

**Table 4:** Pro-fibrotic roles of GSK-3β in various organs/tissues.

Models	Organs/Tissues	Outcomes	References
Male Wistar rats	Heart	Inhibited GSK-3β activity significantly ameliorated cardiac collagen deposition, thereby preventing the development of cardiac fibrosis in diabetic rats.	112
Metallothionein -overexpressing transgenic mice	Heart	Inactivation of GSK-3β significantly represses diabetes-induced cardiac fibrosis by reducing collagen deposition and inflammation.	113
Arrb2 KO mice	Liver	GSK-3β inactivation improves hepatic fibrosis by reducing hepatocyte apoptosis and hepatic injury.	116
C57BL/6 mice	Kidney	Following I/R, GSK-3β increasesα-SMA expression, COLLΙ and FN deposition, and macrophage infiltration.	117
Arrb2 KO mice	Kidney	Activation of GSK-3β increases COLLΙ and FN deposition as well as α-SMA expression, thereby aggravating bilateral IR-induced renal fibrosis.	118
Human lung fibroblasts	Lung	GSK-3β inhibition prevents α-SMA and FN expression, inflammation, and ECM production, and may be beneficial in pulmonary fibrosis.	120
C57BL/6 mice	Lung	Down-regulation of GSK-3β can reverse established mesothelial mesenchymal transition and improve lung function. However, increased GSK-3β expression colocalized with the increased expression of α-SMA and COLLΙ.	121

## Abbreviations

GSK-3β: glycogen synthase kinase-3β
ECM: extracellular matrix
EMT: epithelial-mesenchymal transition
α-SMA: α-smooth muscle actin
SIRT3: sirtuin 3
PI3K: phosphatidylinositol 3-kinase
AKT: protein kinase B
SGK1: serum and glucocorticoid-induced protein kinase 1
TGF-β1: transforming growth factor-β1
PP2A: protein phosphatase 2A
ILK: integrin-linked kinase
PTEN: phosphatase and tensin homolog deleted on chromosome 10
Ser/Thr: serine/threonine
DOCA: deoxycorticosterone acetate
UUO: unilateral ureteral obstruction
COLI: collagen I
COLIII: collagen III
NF-κB: nuclear factor-kappaB
BCL2: B-cell lymphoma 2
Nrf2: Nuclear factor erythroid 2-related factor 2
β-TrCP: β-transducin repeat-containing protein
BECN1: Beclin1
FN: fibronectin
MAPK: mitogen-activated protein kinase
PPARγ: Peroxisome proliferator-activated receptor γ
CNC: cap'n'collar
bZIP: basic-region leucine zipper
OP: polysaccharide isolated from okra
T2DM: type 2 diabetes mellitus
IKK: IκB kinase
ATOR: Atorvastatin
HG: high glucose
*B. cenocepacia*: *Burkholderia cenocepacia*
ROS: reactive oxygen species
GSTA3: Glutathione S-transferase A3
CTSL: Cathepsin L
DM-celecoxib: 2,5-dimethylcelecoxib
GSK-3β^+/-^: GSK-3β heterologous defect
TAC: transverse aortic constriction
Nox2: nicotinamide adenine dinucleotide phosphate oxidase 2
AVICs: aortic valvular interstitial cells
CAVD: calcific aortic valve disease
MI: myocardial infarction
PARP: poly (ADP-ribose) polymerase
IPF: Idiopathic pulmonary fibrosis
PP2A: protein phosphatase 2A
SPARC: secreted protein acidic and rich in cysteine
HSCs: hepatic stellate cells
PDGF: platelet-derived growth factor
Tβ4: thymosin beta-4
CCl_4_: carbon tetrachloride
ILK: linked protein kinase
NO: nitric oxide
NAFLD: nonalcoholic fatty liver disease
ANG II: angiotensin II
UP: urinary protein
sEH: soluble epoxide hydrolase
FSH: follicle‐stimulating hormone
HPMCs: human peritoneal mesothelial cells
GC: Glucocorticoid
MET: mesenchymal-to-epithelial transition
DSS: dextran sulfate sodium
DCM: Diabetic cardiomyopathy
MT-TG: metallothionein
BDL: bile duct ligation
Arrb2: β-arrestin 2
IRI: ischemia-reperfusion injury
HFDs: High-fat diets
SSc: systemic sclerosis
OPN: osteopontin
AF: atrial fibrillation
circRNA: Circular RNA
OS: osteosarcoma
lncRNAs: long noncoding RNAs
